# Serological markers of Bornavirus infection found in horses in Iceland

**DOI:** 10.1186/1751-0147-55-77

**Published:** 2013-11-01

**Authors:** Sigríður Björnsdóttir, Elfa Agustsdóttir, Anne-Lie Blomström, Inga-Lena Örde Öström, Louise Treiberg Berndtsson, Vilhjálmur Svansson, Jonas Johansson Wensman

**Affiliations:** 1 Icelandic Food and Veterinary Authority, Austurvegur 64, IS-800 Selfoss, Iceland; 2 Logmannshild Veterinary Clinic, IS-603 Akureyri, Iceland; 3 Department of Biomedical Sciences and Veterinary Public Health, Swedish University of Agricultural Sciences, P.O. Box 7028, SE-750 07 Uppsala, Sweden; 4 Department of Virology, Immunobiology and Parasitology, National Veterinary Institute, SE-751 89 Uppsala, Sweden; 5 Institute for Experimental Pathology, University of Iceland, Keldur, IS-112 Reykjavik, Iceland; 6 Department of Clinical Sciences, Swedish University of Agricultural Sciences, P.O. Box 7054, SE-750 07 Uppsala, Sweden

**Keywords:** Borna disease, Neurological disease, Serology, Epidemiology, Horse

## Abstract

**Background:**

In a stable of eight horses in Northern Iceland, six horses presented with clinical signs, such as ataxia and reduced appetite, leading to euthanasia of one severely affected horse. Serological investigations revealed no evidence of active equine herpes virus type 1 infection, a common source of central nervous system disease in horses, nor equine arteritis virus and West Nile virus. Another neurotropic virus, Borna disease virus, was therefore included in the differential diagnosis list.

**Findings:**

Serological investigations revealed antibodies against Borna disease virus in four of five horses with neurological signs in the affected stable. One horse without clinical signs was seronegative. Four clinically healthy horses in the stable that arrived and were sampled one year after the outbreak were found seronegative, whereas one of four investigated healthy horses in an unaffected stable was seropositive.

**Conclusions:**

This report contains the first evidence of antibodies to Borna disease virus in Iceland. Whether Borna disease virus was the cause of the neurological signs could however not be confirmed by pathology or molecular detection of the virus. As Iceland has very restricted legislation regarding animal imports, the questions of how this virus has entered the country and to what extent markers of Bornavirus infection can be found in humans and animals in Iceland remain to be answered.

## Findings

Borna disease virus (BDV) is a negative-stranded RNA-virus infecting and causing neurological disease in several warm-blooded animals [1,2]. The clinical signs in horses usually start with disturbances in feed intake, fever and various degrees of somnolence. Later, ataxia, and other gait disturbances, more severe somnolence and finally paralysis of extremities and head develop (reviewed in [2]). Other mental changes are also common, such as depression, coma and excitations. The virus transmission routes are still obscure, but the involvement of reservoir hosts, such as wild birds, rodents and insectivores, has been proposed [1,3-5]. Most cases of equine Borna disease (BD) have been reported from Central Europe; however, BDV-infection markers have been reported all over the world [1]. Avian Bornavirus (ABV) was recently found to cause proventricular dilatation disease in psittacine and other avian species, increasing the host spectrum and spread of these intriguing viruses [1-3].

Here, we describe the first evidence of antibodies to BDV in Iceland, detected in an outbreak of neurological disease in horses.

In a geographically isolated fishing village in northern Iceland there is a cluster of twelve stables that house about 70–80 horses during the winter. In February 2011, one of the stables (stable A) with 8 horses reported neurological signs in horses (for details see Table 1). The clinical signs started in a 21-year-old gelding (horse no. 1) that presented with pelvic limb ataxia and reduced appetite. Normal body temperature and full consciousness was recorded at first day of clinical signs (day 0) in this horse. After 3–4 days of treatment (Table 1), the horse (no. 1) improved temporarily; however, after another week it developed more severe ataxia and loss of appetite, finally becoming paralysed in its pelvic limbs and was euthanized (day 21). At this time-point (day 13–14), five other horses (horses no. 2–6) were also affected, showing various degrees of pelvic limb ataxia, depression or excitation and reduced appetite (Table 1). The body temperature of all horses was found to be within the normal range. As the horses were examined under field conditions, a detailed neurological status was, however, not achieved. All horses, except for one mare (horse no. 8) that showed no clinical signs and had arrived 10 days before the time of onset of clinical signs in horse no. 1 (day −10), were treated as indicated in Table 1. Horses no. 5–6 recovered by day 21, while horses no. 2–4 had a slow recovery and were only fully recovered around 3 months after onset of signs in the index case. Horse no. 2 was euthanized approximately 6 months after onset of signs due to age and a history of intermittent lameness, most likely independent of the neurological signs. Horse no. 8 did not show any clinical signs throughout the whole observation period (until day 440).

**Table 1:** Clinical signs and history of horses in the affected stable (stable A)

**Horse ID/age**	**Day 0**	**Day 7**	**Day 14**	**Day 21**
1.	Ataxia and reduced appetite	Temporary improvements	Severe ataxia/paralysis and loss of appetite	Euthanized
21 y	Treatment 1^a^ at day 4		Treatment 2^b^	
2.^c^	No clinical signs	No clinical signs	Ataxia, depression and reduced appetite	Slow recovery
21 y			Treatment 2^b^	
3.	No clinical signs	No clinical signs	Ataxia, mild excitation and reduced appetite	Slow recovery
12 y			Treatment 2^b^	
4.	No clinical signs	No clinical signs	Ataxia, mild excitation and reduced appetite	Slow recovery
18 y			Treatment 2^b^	
5.	No clinical signs	No clinical signs	Mild signs of ataxia and excitation	Recovery
13 y			Treatment 2^b^	
6.	No clinical signs	No clinical signs	Mild signs of ataxia	Recovery
6 y			Treatment 2^b^	
7.	No clinical signs	No clinical signs	No clear signs	Recovery
9 y			Treatment 2^b^	
8.	Arrived to the stable 10 days earlier. No clinical signs	No clinical signs	No clinical signs	No clinical signs

^a^Treatment 1 is dexamethasone 20 mg/day for 6 days i.m., procaine benzyl penicillin 4000 mg/day for 6 days i.m.

^b^Treatment 2 is oxytetracycline 4000 mg i.v. once, glucose 30% 1000 ml i.v. once, metamizole 1500 mg i.v. once, dexamethasone 20 mg/day i.m. daily for 8 days, ampicillin 1.67 g/day i.m. daily for 6 days, with additional support: medical coal p.o. daily for 8 days, Prolac AB p.o. daily for 6 days, Pro-Bran (Protexin, Probiotics, Somerset, UK) p.o. daily for 12 days, Ferro Complex (Blue Hors, Randbøl, Denmark) p.o. for 1 month.

^c^This horse was euthanized approx. 6 months after the onset of signs, because of age and a history of intermittent lameness, most likely independent of the neurological signs.

Initially, serum samples from four of the horses (horses no. 2, 3, 5 and 6) were taken for serological analyses 9–10 days after they presented with neurological signs and three weeks after onset of signs in the index case (day 23). No antibodies towards equine herpes virus (EHV) types 1 and 4 were found by complement fixation. Presence of IgG-antibodies against EHV-1 detected by ELISA (Svanovir EHV1/EHV4-Ab, Svanova, Uppsala, Sweden) in one horse (no. 5) was not regarded to indicate an active EHV-1 infection and none of the other investigated horses in the outbreak stable had this finding in the observation period (from day 23 to 440). Antibodies to equine arteritis virus (EAV) and West Nile virus (WNV) were not detected by serum neutralization test and ELISA (ID Screen West Nile Competition, ID-vet, Montpellier, France), respectively. Infection with an alphavirus was considered unlikely based on the clinical signs and epidemiology. We therefore decided to include BDV as a differential diagnosis.

An indirect immunofluorescence assay (IFA) was employed as previously described [6], except for the use of fluorescein isothiocyanate (FITC) conjugated anti-horse IgG antibodies as secondary antibodies. Horse sera previously characterised as positive or negative for BDV antibodies in a BDV-ELISA were used as controls, whereof one of the positive controls also was PCR-positive and positive at Western blotting [7]. Serum incubation on slides with non-infected cells was used as negative controls, to exclude the possibility for unspecific fluorescence signals due to cross-reactivity. All serum samples were sent for a second IFA analysis [8] (Idexx/Vet Med Labor, Ludwigsburg, Germany). An individual sample was considered seropositive if a titre of ≥1:40 was detected in both IFAs, negative if both titres were <1:40, and doubtful if only one of the IFAs showed a titre of ≥1:40.

Around 10 days after onset of clinical signs, all horses showing clinical signs (horses no. 2–5) were positive in the first IFA, and two of the horses (horses no. 2 and 5) were positive by the second IFA, and thus defined seropositive (Table 2). These results led to an increased monitoring of the horses in the affected stable (Tables 2 and 3). Blood and serum samples were collected 380–440 days after the outbreak from 3 of the 4 horses initially tested. Additionally, blood samples were collected from 6 other horses in stable A. Three of these 6 horses had been in the stable at the time of the outbreak (Tables 2 and 3). The horses were either seropositive (no. 2, 3, 5–7) or doubtful seropositive (no. 4) for BDV-antibodies (Table 2). No BDV-specific antibodies were detected in horse no. 8 that arrived to the stable at day −10, indicating that BDV entered the stable before that time. This horse showed no clinical signs throughout the observation period (until day 440). Likewise, all horses, that were housed in stable A in 2012 but not in 2011, did not show any signs of neurological disease and were seronegative (Table 3). As comparison, horses in another stable (stable B) with no evidence of neurological disease, situated approximately 50 km southwest of the affected stable, were investigated. In stable B one horse had presence of BDV-specific antibodies, one was seronegative and two were considered doubtful (Table 3). There were no contacts between stables A and B.

**Table 2:** BDV serology of horses in the affected stable A

**Horse ID**	**Clinical signs**^**a **^**(see Table**1**)**	**Day 23 (IFA1**^**b**^**/IFA2**^**c**^**)**	**Approx. day 380 (IFA1**^**b**^**/IFA2**^**c**^**)**	**Approx. day 440 (IFA1**^**b**^**/IFA2**^**c**^**)**^**d**^
1.	++++	n.d.^e^	n.d.	n.d.
2.	++++	1:160/1:80	n.d.	n.d.
3.	++	1:80/<1:10	1:40/<1:10	1:160/1:40
4.	++	n.d.	n.d.	<1:20/1:40
5.	+	1:160/1:40	1:320/<1:10	1:160/<1:10
6.	+	1:80/<1:10	1:160/1:40	n.d.
7.	(+)	n.d.	n.d.	1:160/1:40
8.	-	n.d.	n.d.	<1:20/<1:10

^a^The degree of clinical signs is indicated as ++++ = severe clinical signs, ++ = moderate signs, + = mild signs, and (+) = no clear signs.

^b^IFA1 is the IFA performed in Sweden.

^c^IFA2 is the IFA performed in Germany.

^d^Samples were blindly tested.

^e^n.d. indicates not done.

**Table 3:** BDV serology of clinically healthy horses

**Horse ID/Stable ID**	**IFA1**^**a**^**/IFA2**^**b**^
9. / A-12	<1:20/<1:10
10. / A-12	<1:20/<1:10
11. / A-12	<1:20/<1:10
12.^c^ / B	1:160/<1:10
13.^c^ / B	<1:20/<1:10
14.^c^/ B	1:160/1:160
15.^c^/ B	1:40/<1:10

^a^IFA1 is the IFA performed in Sweden.

^b^IFA2 is the IFA performed in Germany.

^c^Samples were blindly tested.

A-12 refers to horses that entered the affected stable the year after the outbreak (2012). Stable B is situated ca. 50 km southwest of stable A. The two stables have had no contacts.

Molecular diagnostics was performed using previously described real-time and conventional RT-PCR assays targeting three genes of BDV (P, N and M) [6,9,10]. Total RNA was extracted from peripheral blood drawn at day 380 from horses no. 3, 5 and 6 as previously described [6]; however, we could not confirm presence of BDV-RNA in any of the horses.

The euthanized horses were not sent for necropsy; thus, there are no pathology data confirming inflammatory changes of the central nervous system indicating virus infection. Nevertheless, this report shows the first evidence of antibodies to BDV in Iceland, a country with extremely strict animal import regulations, indicating that these horses have been exposed to BDV or a closely related virus. In 1998, we conducted a study of 17 Icelandic horses with pyrexia of unknown reason, which found all to be seronegative by BDV-ELISA (performed at Robert Koch Institute, Berlin, Germany). How the virus has entered Iceland remains to be determined. One possible route of transmission is by migratory birds, because wild birds have been indicated as potential reservoirs of BDV [1,3]. A unique strain of Bornavirus was recently found in healthy Canada geese in the USA, indicating that this migrating waterfowl could carry virus over long distances [11]. Around 60 species of migrating birds visit Iceland during April to October. Many of them pass North Iceland, including swans, geese and loons. The Canada goose is recognised as an annual visitor in spring and autumn, originating both from Europe and North America [12]. Because of the extremely rigid animal import regulations of Iceland, migratory birds are more likely routes of Bornavirus transmission, than domestic animal transports. It has also been proposed that horses in stables along the paths of migratory birds have higher seroprevalence than horses in areas absent of migratory birds [13]. As antibodies to both BDV and ABV cross-react with the strain (He/80) used in the IFAs in our study [14], we cannot exclude that the virus circulating in Iceland could be ABV or a more avian-like Bornavirus.

In conclusion, we present the first evidence of antibodies to Bornavirus in Iceland, found in horses with and without neurological signs. The aetiology of the neurological signs has not been confirmed, and other causes, such as toxins or other viruses, cannot be excluded. Acquired equine polyneuropathy has recently been described in Swedish, Norwegian and Finnish horses, but this syndrome does not give rise to ataxia [15]. Hence, it seems unlikely that the horses in this study suffered from this syndrome. The questions of how Bornavirus has entered the country and to what extent markers of Bornavirus infection can be found in humans and animals in Iceland remain to be answered.

## Abbreviations

ABV: Avian Bornavirus; BD: Borna disease; BDV: Borna disease virus; EAV: Equine arteritis virus; EHV: Equine herpes virus; ELISA: Enzyme-linked immunosorbent assay; FITC: Fluorescein isothiocyanate; IFA: Immunofluorescence assay; IgG: Immunoglobulin G; RT-PCR: Reverse transcription polymerase chain reaction; WNV: West Nile virus.

## Competing interests

The authors declare that they have no competing interests.

## Authors’ contributions

SB conceived and designed the study, performed sampling, clinical evaluation, interpreted the data and helped to draft the manuscript. EA performed sampling, clinical examinations and treatment. ALB performed molecular diagnostics and helped to draft the manuscript. ILÖ performed and analysed serological assays. LTB participated in the conception and design of the study, and helped to draft the manuscript. VS conceived and designed the study, interpreted the data, and helped to draft the manuscript. JJW conceived, designed and coordinated the study, performed and analysed serological assays, interpreted the data and drafted the manuscript. All authors approved the final manuscript.
